# Management of Inedible Airway Foreign Bodies in Pediatric Rigid Bronchoscopy: Experience From a National Children's Regional Medical Center in China

**DOI:** 10.3389/fped.2022.891864

**Published:** 2022-06-22

**Authors:** Bin Xu, Lei Wu, Jing Bi, Jia Liu, Cao Chen, Lexi Lin, Chao Chen, Fei Qiu, Shiqiang Shang

**Affiliations:** ^1^Department of Otorhinolaryngology-Head and Neck Surgery, Department of Endoscopy Center, The Children's Hospital, Zhejiang University School of Medicine, National Clinical Research Center for Child Health, National Children's Regional Medical Center, Hangzhou, China; ^2^Department of Pulmonology, Department of Endoscopy Center, The Children's Hospital, Zhejiang University School of Medicine, National Clinical Research Center for Child Health, National Children's Regional Medical Center, Hangzhou, China; ^3^Department of Radiology, The Children's Hospital, Zhejiang University School of Medicine, National Clinical Research Center for Child Health, National Children's Regional Medical Center, Hangzhou, China; ^4^Department of Clinical Laboratory, The Children's Hospital, Zhejiang University School of Medicine, National Clinical Research Center for Child Health, National Children's Regional Medical Center, Hangzhou, China

**Keywords:** foreign bodies, inhalation, inedible, bronchoscope, child

## Abstract

The aim of this study is to analyze the characteristics of inedible airway foreign bodies in pediatric rigid bronchoscopy to facilitate the improvement of management and technology. This retrospective analytical study was performed from January 2017 to June 2020. All admissions of pediatric patients (age<18 years) with foreign-body aspiration diagnosis codes ([ICD]-10:T17 300, T17 400, T17 500 and T17 900) and procedure codes (33.7801) were extracted. Age, sex, preoperative history and imaging data, surgical records, length of hospital stay, reoperations and postoperative complications were included. Data were analyzed with SPSS 20. A total of 1237 patients were hospitalized and underwent rigid bronchoscopy. Forty-five (3.6%) patients with inedible foreign bodies in the airway were confirmed. There were no significant differences in sex, time of onset and length of hospital stay between the inedible and edible foreign body groups, except for age and a definite history of foreign body aspiration (*P* = 0.000). Coughing, wheezing and fever were the common clinical symptoms in all patients. The following were the common locations of inedible foreign bodies: right bronchus (22/45), left bronchus (18/45), trachea (3/45) and larynx (2/45). The most frequent inedible foreign bodies were parts of a pen (15/45), a light-emitting diode (7/45) and plastic parts of toys (6/45). Vocal cord injury and a laryngeal web were observed in one case each.

**Conclusion:**

Rigid bronchoscopy is the method of choice for the removal of inedible foreign bodies. Adequate preoperative assessment to rely on CT scans, skillful operation techniques to avoid damaging and active management of postoperative complications are important for the success of the procedure.

## Introduction

Airway foreign bodies are a common cause of morbidity and mortality in pediatric patients, especially in young infants. Foreign body aspiration is a serious condition and requires immediate management to avoid irreversible lung injury (1). It can be associated with severe complications in children, even cardiopulmonary arrest and sudden death. The age of children with airway foreign bodies is mostly <5 years, and the in-hospital mortality rate ranges from 0.36 % to 2.75%, as previously reported (2–4). Apart from age, airway foreign bodies were also related with male sex, lack of insurance and geographical location (3).

Rigid bronchoscopy under general anesthesia is the gold standard for diagnosis and treatment of airway foreign body (5). Allowing for ongoing ventilation throughout in rigid bronchoscopy provides airway security and sufficient time to remove foreign bodies (6). Using grasping forceps can make the operation more efficient. These features are rigid bronchoscopy's distinct advantage during foreign-body retrieval. Nevertheless, removal of a foreign body from a pediatric airway is undoubtedly a hard-fought battle. Due to their small airways, the lack of a visual field and working channels makes the management in pediatric bronchoscopes more complicated and challenging (7). Most aspirated foreign bodies in children are food-related, mainly fragments of seeds and nuts (8, 9). Residual foreign bodies in the airway are a troublesome problem in rigid bronchoscopy, as shown in our recent report (10). However, there are still a few inedible foreign bodies in the airway that are challenging to doctors and patients, and this problem has not been well described in the literature.

Here, we review our experience with inedible foreign bodies in procedure with rigid bronchoscopy to facilitate the improvement of management and technology.

## Materials and Methods

The data source for this study consisted of The Children's Hospital, Zhejiang University School of Medicine (National Clinical Research Center for Child Health, National Children's Regional Medical Center) from January 2017 to June 2020. The study was reviewed and approved by the Ethics Committee of the Children's Hospital, Zhejiang University School of Medicine. For each year, all admissions of pediatric patients (age<18 years) with foreign-body aspiration diagnosis codes (International Classification of Diseases [ICD]-10 diagnosis codes: T17 300, T17 400, T17 500 and T17 900) and procedure codes (bronchoscopy with foreign body removal: 33.7801) were extracted from the ENT department. Age, sex, preoperative history and imaging data, surgical records, length of hospital stay, reoperations and postoperative complications were included in this study.

The data were analyzed with SPSS 20 using the rank-sum test and chi-square test to compare the values of between the specific study groups.

## Results

This retrospective analytical study included 1237 patients who were hospitalized in our hospital and underwent rigid bronchoscopy to diagnose and remove foreign bodies in the airway. There were 810 boys and 427 girls, ranging from 6 months (m) to 13 years (y) of age. The mean age was 1.93 (interquartile range, IQR: 1.24, 2.03) years. Forty-five patients with inedible foreign bodies in the airway were confirmed by rigid bronchoscopy. The proportion of inedible airway foreign bodies was 3.6%. The mean age was 5.22 (IQR: 1. 22, 8.84) years. There were 33 boys (73.3%) and 12 girls (27.7%), with a male/female ratio of 2.75:1. The time of onset before admission was 8.78 (IQR: 0.36, 7.00) days. There was no significant difference in sex, time of onset and length of hospital stay between the inedible and edible groups (Table 1). There was significant difference in age. Thirty-three patients over 3 years old accounted for 73.3% of the inedible group, and 1103 patients <3 years old accounted for 92.5% of the edible group.

**Table 1:** Comparison of age, sex, time of onset and length of hospital stay between the edible foreign body group and the inedible group.

	***N***	**Age (year)^**Δ**^, mean (IQR)**	**Sex (male/female) ^**▴**^**	**Time of onset (day) ^**Δ**^, mean (IQR)**	**Length of hospital stay (day) ^**Δ**^, mean (IQR)**
Edible group	1192	1.8(1.24,1.97)	777/415	6.15(0.50,5.00)	4.01(3.00,5.00)
Inedible group	45	5.22(1.22,8.84)	33/12	8.78(0.36,7.00)	4.11(3.00,5.00)
Statistic		4.860	1.274	0.066	1.002
*P*		0.000^*^	0.259	0.947	0.316

*IQR, interquartile range; Δ, Rank sum test; ▴, Chi-square test*.

* *P < 0.05*

Thirty-seven patients had a definite history of foreign body aspiration in the inedible group and 1138 patients in the edible group, and there was a significant difference (*P* = 0.000). Seven cases (7/8) without a history of foreign body aspiration in the inedible group were <5 years old, and their average age was 2.67 years (range 1.07 to 4.66 years).

Coughing (97%), wheezing (89.7%) and fever (9.9%) were the common clinical symptoms in all patients (Table 2). Most patients (98.7%) underwent chest spiral CT scans and 4.2% underwent chest X-ray films before the operation. As a result, the imaging examination of 37.3% of the cases demonstrated pneumonia or bronchitis following aspiration of airway foreign bodies. Mediastinal emphysema occurred in three cases in the inedible group and thirteen in the edible group. One case in each group had pneumothorax.

**Table 2:** Clinical features of the edible foreign body group and the inedible group.

	**Edible group**	**Inedible group**
*N*	1192	45
Symptom		
Coughing	1162	38
Wheezing	1075	35
Fever	117	6
Cyanosis	73	6
Hoarseness	39	5
Laryngeal stridor	28	1
Preoperative examination		
CT	1177	44
Chest X-ray	50	2
Bronchoscope	8	0
None	4	0
Imaging results		
Inflammation	411	13
Mediastinal emphysema	13	3
Pneumothorax	1	1
Postoperative complications		
Residual foreign body	17	0
Traumatic laryngeal web	0	1
Vocal cord damage	0	1

**Figure 1:**
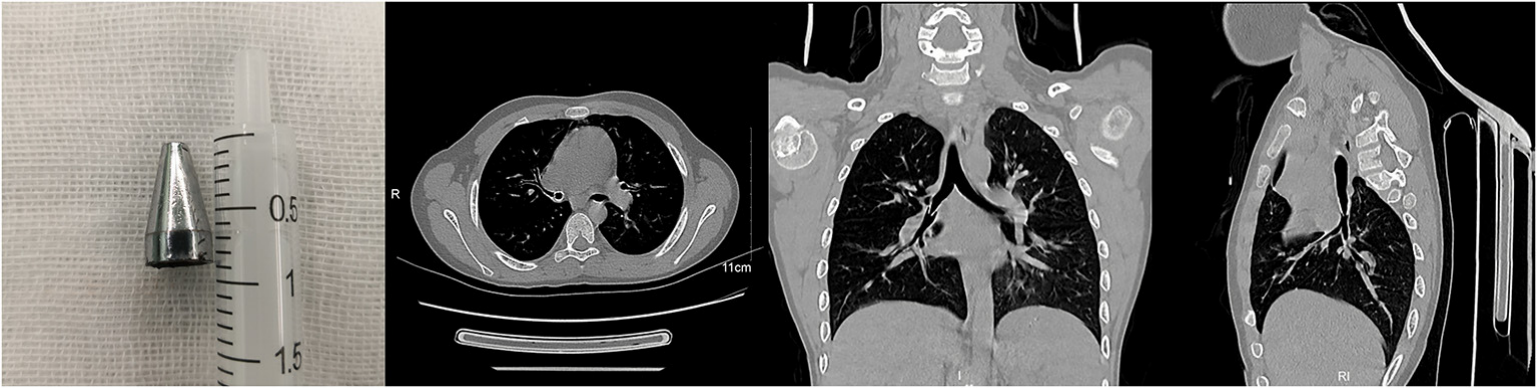
A stand of a pen in the right main stem bronchus of a nine-year-old boy.

**Figure 2:**
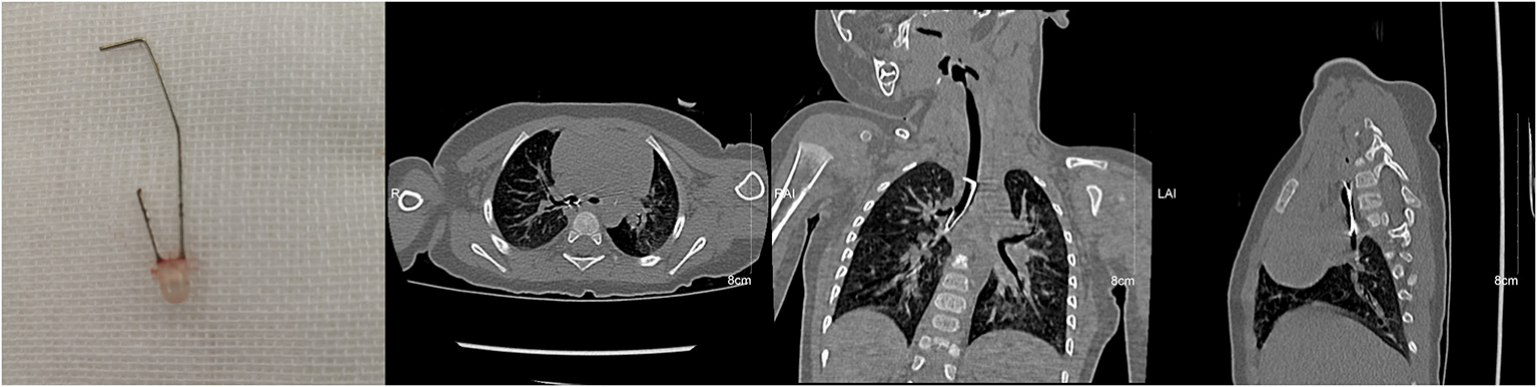
A light-emitting diode in the right main stem bronchus of a nine-month-old girl.

**Figure 3:**
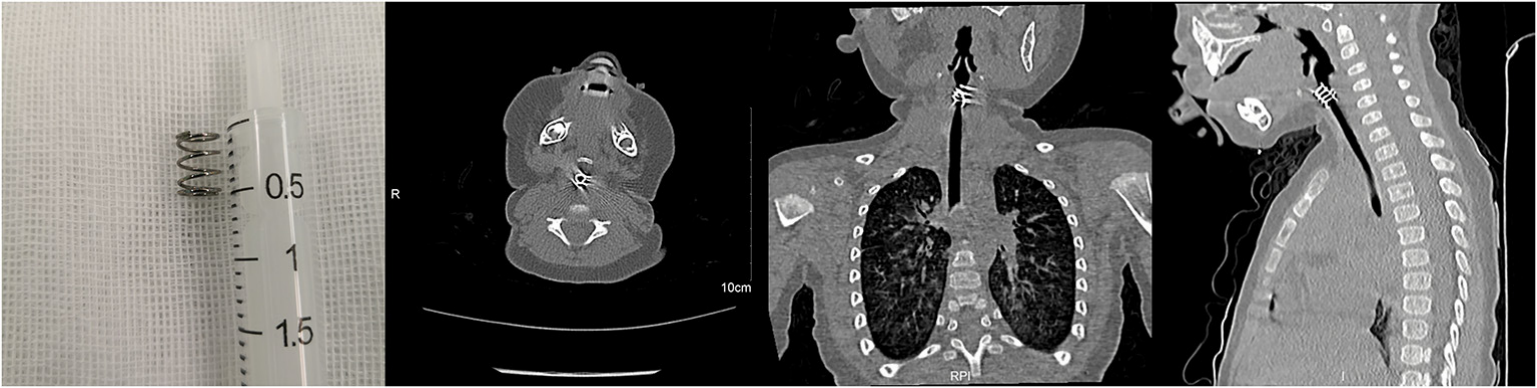
A spring in the glottis of a nine-month-old boy.

**Figure 4:**
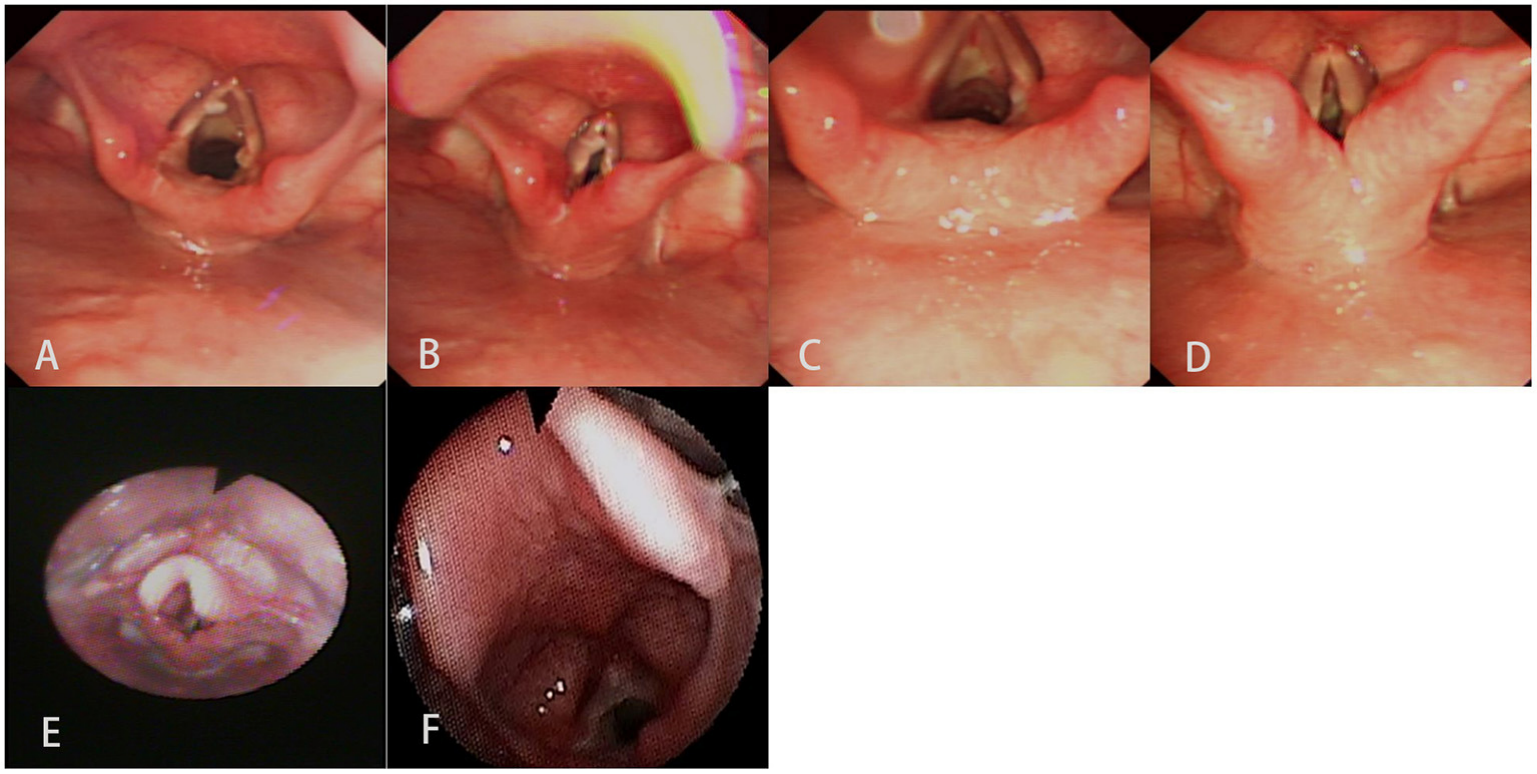
Electronic laryngoscopy showed that there was vocal cord injury in an eight-year-old boy who aspirated a plastic cap of a pen. **(A,B)** Seven days after rigid bronchoscopy. **(C,D)** Half month after rigid bronchoscopy. A fiberoptic laryngoscope showed that there was a laryngeal web in a nine-month-old boy who aspirated a spring. **(E)** Five days after rigid bronchoscopy (without the laryngeal web). **(F)** Six months after rigid bronchoscopy (with the laryngeal web).

The rigid bronchoscopy procedure was performed by pediatric otolaryngologists. It was found that the common locations of inedible foreign bodies were as following: right bronchus (22/45), left bronchus (18/45), trachea (3/45) and larynx (2/45) (Table 3). The most frequent inedible foreign bodies were parts of a pen (15/45) (Figure 1), light-emitting diodes (7/45) (Figure 2) and plastic parts of toys (6/45) (Table 4). Most metallic foreign bodies were specific and sharp-pointed, such as light-emitting diodes, reeds, springs (Figure 3), brooches, screws, thumbtacks and nails. Vocal cord injury occurred during the rigid bronchoscopy procedure for an eight-year-old boy who aspirated a plastic cap of a pen but he fully recovered half a month after foreign body removal (Figures 4A–D). A 9-month-old boy aspirated a spring into the glottis, and a laryngeal web was found 6 months after its removal (Figures 3, 4E,F). There were no mortalities noted in this cohort.

**Table 3:** Location of foreign bodies in the airway between the edible foreign body group and the inedible group.

	**Edible group**	**Inedible group**
Larynx	5	2
Trachea	88	3
Bronchus		
Left	535	18
Right	549	22
Bilateral	15	0

**Table 4:** Type of foreign bodies in the airway between the edible foreign body group and the inedible group.

**Edible group**			**Inedible group**		
Nuts			Plastic		
Peanut	463		Pen		
Wild walnut kernel	124		Cap	10	
Chestnut	46		Stand	5	
Pistachio nuts	25		Plastic part of toys	6	
Almond	19		Beads	2	
Pine nuts	18		Press of lighter	1	
Seeds			Plastic wrap	1	
Sunflower seeds	131		Metal		
Watermelon seeds	66		Light-emitting diode	7	
Pumpkin seeds	49		Tinfoil	3	
Mongolian snake gourd seed	12		Reeds	1	
Beans			Spring	1	
Soybean	27		Button	1	
Broad bean	14		Brooch	1	
Others nuts, seeds & beans	91		Screw	1	
Flesh of unclear nature	41		Thumbtack	1	
Shell of unclear nature	13		Nails	1	
Bone slice	46		Tooth	2	
Unclear in nature	7		Unclear in nature	1	

## Discussion

Approximately 2000 pediatric patients undergoing foreign body aspiration are hospitalized annually in the United States, and the median length of stay is 3 (IQR: 1, 7) days (11). There was no exact data available about morbidity and mortality in Chinese children who inhale foreign bodies because of the huge population base and owing to lack of a unified medical database. In our study, we found that coughing, wheezing, fever and cyanosis were the major clinical features in children who underwent inedible foreign body aspiration. Males were the majority, as expected. The mean age was 5.22 years in the inedible group and 1.80 years in the edible group, which was due to the characteristics of customary behavior in different age groups.

Parts of a pen (cap and stand, 33.3%), light-emitting diodes (15.6%) and plastic parts of toys (13.3%) constituted the majority of inedible foreign bodies, which affected 3.6% of all patients. An Indian study showed that 15.3% of patients had non-vegetative foreign bodies, and whistles (45.4%), pen caps (36.36%) and stones (18.2%) were the most common retrieved objects in these cases (12). Jiaqiang S et al. reported that pen cap inhalation was 2.65% of all cases, most frequently found in patients aged 6 to 14 years, and 76.4% of them were in the right main stem bronchus (13). Our result showed that most (86.7%) cases of pen aspiration occur in school age children (over 6 years old) because students are accustomed to placing pens in their mouth and then inhaling the objects when talking or laughing (14). 85.7% in cases of light-emitting diodes and 83.3% in cases of plastic parts of toys were accidents in preschool children due to their playful activity and propensity to bite toys. Similarly, another study reported that parts of ballpoint pens, toys, plastics and pendants account for nearly seventy percent of inorganic substances of foreign body aspiration in childhood (15).

### Diagnosis/Work up

Because of the large volume and irregular shape of the inedible foreign bodies, the difficulty and risk of the operation were significantly higher than those of the edible group. Therefore, adequate preoperative preparation was especially important for a successful operation. CT scans could be a helpful means to evaluate the condition before surgery, especially in potentially high-risk cases (16). Seventeen cases of foreign bodies were related to metals, and two were teeth. These could be identified by obtaining high-density images on CT scans. CT is effective for diagnosing airway foreign bodies, with a sensitivity of 100% and a specificity of 98% (17). Meanwhile, CT scans are also helpful for identifying the presence of complications such as mediastinal emphysema and pneumothorax, which might lead to unstable conditions. The incidence of mediastinal emphysema secondary to foreign bodies was 1.5%, and the incidence of pneumothorax was 0.4% (18). The mortality can reach 5.1% if these emergencies are not recognized (18).

Meanwhile, CT scans are advantageous to delineate the exact shape, location and volume of the foreign body to evaluate the surgical risk and to formulate a surgical strategy for safe removal of the foreign body (19). However, some foreign bodies have only some metal parts, or could not be shown as high-density shadows; for example, light-emitting diodes with lamp beads and other objects such as plastic wrap, so it is important to know the history of the case before surgery. Asking about the history is very important and must not be ignored in any case (20). At the same time, we need to make a backup plan and prepare for tracheotomy to prevent asphyxia.

### Operative Technique

How to grasp the optimal position conducive to the removal of foreign bodies was the first step. It should be based on careful reference to imaging data and previous surgical experience. Grasping the edge of the foreign body, such as the cap of a pen, after taking the hollow end face upward would make removal easier. If the foreign body is stuck too tightly to catch it, bronchoscopic cryoprobe extraction might be a safe and effective option. A previous study (21) made attempts in four cases, and was successful in half. Another study also described a case using cryotherapy to remove aspirated sponges from an adult patient (22). The tip of the probe could freeze the liquid within or surrounding the foreign object (i.e., metal or plastic), which could cause it to be reliably adherent, with the aim of cryoadherence and extraction (23). Our experience with foreign body cryoextraction is limited, and we tried this method in an 8-year-old boy with a pen cap in the right main bronchus that failed. For sharp objects, the sharp part should be hidden in the forceps or in the bronchoscopic tube to avoid damaging the bronchial tissue.

The glottis is the narrowest portion of the airway in children aged 6 months to 13 years under general anesthesia (24), which makes it difficult for foreign bodies to pass through. Endoscopy showed that the vocal cords were in an adducted position in a 1-year-old child who spontaneously breathed under inhalation anesthesia (25). The object lodged below the vocal cords is dangerous and could cause dyspnea at any time, such as a board game piece or a pen cap (14). When an unusually shaped foreign body directly passes through the vocal folds and completely obstructs the respiratory tract, particularly in children, the patients undergo choking episodes and lie between life and death (26). If forceps cannot be used to remove the object immediately, the object should be moved into the right mainstem bronchus naturally between attempts. The position of the sharp part, such as that on a light-emitting diode or thumbtack, should be adjusted or confirmed again when crossing the glottis to avoid damaging the vocal cords. When rigid bronchoscopy fails because objects are unable to pass through the glottis, tracheotomy has to be performed, and the wound could be sutured immediately after the foreign body is removed (27). Other literature reported a tracheostomy rate of 1.2% (28).

### Complications

The complications of rigid bronchoscopy mostly manifest as laryngeal edema, injury to the vocal cords, airway laceration and perforation, hypoxemia-induced cardiac ischemia and arrhythmias (29). Vocal cord injury from rigid bronchoscopy includes mucosal lacerations and laceration of the free edge, leading to prolonged recovery and long-term dysphonia as sequelae (30). Interventions to minimize scar formation are vital for optimizing phonatory function. There were two cases of vocal cord injury in our series of cases, and the incidence was 0.16% of all (2/1237). Both of them were in the inedible group. The 8-year-old boy lost his voice when he underwent a lengthy procedure to remove a pen cap that was broken during the operation. Laryngoscopy showed that the mucosa of the vocal cords was injured, but he fully recovered after a period of recuperation. The 9-month-old boy had hoarseness after the spring in his glottis was removed, but it was irrelevant to iatrogenic injury. The traumatic laryngeal web was not found until fiberoptic laryngoscopy was performed 6 months later, and we have not yet found a better way to solve this issue. A laryngeal web is a challenging surgical issue, and bilateral injured adjacent mucosa on the anterior vocal cords increases the risk of laryngeal web recurrence (31). Keel placement or laryngeal stenting might optimize these surgical techniques.

In our series, there was no mediastinal emphysema or pneumothorax secondary to the procedure, only those caused by the foreign body itself. Pneumothorax as a postoperative complication is rare, with an incidence of 0.3% (32). For children without dyspnea or with mild dyspnea, pneumothorax could be managed conservatively (33). Most of them resolve spontaneously in a few days. Pneumothorax followed by moderate or severe dyspnea should be treated by pleural cavity drainage immediately, and the patient's vital signs should be carefully monitored. In all, serious consequences could be avoided as long as complications are handled in a timely manner.

## Conclusion

In summary, inedible airway foreign bodies are uncommon but challenging problems. Rigid bronchoscopy is the gold standard technique and procedure for the management of inedible foreign body aspiration. Patients need to be fully evaluated to depend on CT scanning before surgery, and the procedures have to be more tailored to the intraoperative details to avoid injuring the airway mucosa. Postoperative complications also should be actively considered. This study was limited to one medical center, and a multicenter study should be conducted.

## Data Availability Statement

The raw data supporting the conclusions of this article will be made available by the authors, without undue reservation.

## Ethics Statement

The studies involving human participants were reviewed and approved by the Ethics Committee of the Children's Hospital, Zhejiang University School of Medicine (2020-IRB-192). Written informed consent to participate in this study was provided by the participants' legal guardian/next of kin.

## Author Contributions

BX contributed to conception, design, acquisition, analysis, and interpretation of data. LW and JB contributed to acquisition and analysis of data. JL, CaC, LL, ChC, and FQ contributed to acquisition of data. SS contributed to conception, design, critical revision, and final approval of data. All authors contributed to the article and approved the submitted version.

## Conflict of Interest

The authors declare that the research was conducted in the absence of any commercial or financial relationships that could be construed as a potential conflict of interest.

## Publisher's Note

All claims expressed in this article are solely those of the authors and do not necessarily represent those of their affiliated organizations, or those of the publisher, the editors and the reviewers. Any product that may be evaluated in this article, or claim that may be made by its manufacturer, is not guaranteed or endorsed by the publisher.

## References

[1] Hitter A Hullo E Durand C. Diagnostic value of various investigations in children with suspected foreign body aspiration: review. Eur Ann Otorhinolaryngol Head Neck Dis. (2011) 128:248–52. 10.1016/j.anorl.2010.12.011 22018977

[2] Tan GX Boss EF Rhee DS. Bronchoscopy for pediatric airway foreign body: thirty-day adverse outcomes in the ACS NSQIP-P. Otolaryngol Head Neck Surg. (2019) 160:326–31. 10.1177/0194599818800470 30226798

[3] Cheng J Liu B Farjat AE. National estimations of airway foreign bodies in children in the United States, 2000 to 2009. Clin Otolaryngology. (2019) 44:235–39. 10.1111/coa.13261 30450702 PMC6488414

[4] Johnson K Linnaus M Notrica D. “Airway foreign bodies in pediatric patients: anatomic location of foreign body affects complications and outcomes”. Pediatr Surg Int. (2017) 33:59–64. 10.1007/s00383-016-3988-9 27738825

[5] Li P Jiang G Li Q. “The risks of postoperative complications and prolonged hospital stay in children receiving bronchoscopy”. J Pediatr Surg. (2020) 55:1309–12. 10.1016/j.jpedsurg.2019.05.014 31171352

[6] Londino AR Jagannathan N. “Anesthesia in diagnostic and therapeutic pediatric bronchoscopy”. Otolaryngol Clin North Am. (2019) 52:1037–48. 10.1016/j.otc.2019.08.005 31521368

[7] Na”Ara S Vainer I Amit M. “Foreign body aspiration in infants and older children: a comparative study”. Ear Nose Throat J. (2020) 99:47–51. 10.1177/0145561319839900 30974996

[8] Sink JR Kitsko DJ Georg MW. “Predictors of foreign body aspiration in children”. Otolaryngol Head Neck Surg. (2016) 155:501–7. 10.1177/0194599816644410 27071446

[9] Mohammad M Saleem M Mahseeri M. “Foreign body aspiration in children: a study of children who lived or died following aspiration”. Int J Pediatr Otorhinolaryngol. (2017) 98:29–31. 10.1016/j.ijporl.2017.04.029 28583498

[10] Xu B Wu L Jin Z. “Residual airway foreign bodies in children who underwent rigid bronchoscopy”. Int J Pediatr Otorhinolaryngol. (2019) 118:170–76. 10.1016/j.ijporl.2019.01.007 30639987

[11] Kim IA Shapiro N Bhattacharyya N. “The national cost burden of bronchial foreign body aspiration in children”. Laryngoscope. (2015) 125:1221–24. 10.1002/lary.25002 25363312

[12] Chouhan M. Sharma S. “Tracheobronchial Foreign Bodies: The Importance of Timely Intervention and Appropriate Collaboration”. Indian J Otolaryngol Head Neck Surg. (2019) 71 (Suppl. 1):972–5. 10.1007/s12070-019-01659-1 31742104 PMC6848424

[13] Jiaqiang S Jingwu S Yanming H. “Rigid bronchoscopy for inhaled pen caps in children”. J Pediatr Surg. (2009) 44:1708–11. 10.1016/j.jpedsurg.2008.11.035 19735812

[14] Samra S Schroeder JJ Valika T. “Tracheotomy for difficult airway foreign bodies in children”. Otolaryngol Head Neck Surg. (2018) 158:1148–9. 10.1177/0194599818758995 29437526

[15] Rodriguez H Cuestas G Botto H. “Complications in children from foreign bodies in the airway”. Acta Otorrinolaringol Esp. (2016) 67:93–101. 10.1016/j.otoeng.2016.03.006 25857247

[16] Reid A Hinton-Bayre A Vijayasekaran S. “Ten years of paediatric airway foreign bodies in Western Australia”. Int J Pediatr Otorhinolaryngol. (2020) 129:109760. 10.1016/j.ijporl.2019.109760 31751807

[17] Gibbons AT Casar BA Hanke RE. “Avoiding unnecessary bronchoscopy in children with suspected foreign body aspiration using computed tomography”. J Pediatr Surg. (2020) 55:176–1. 10.1016/j.jpedsurg.2019.09.045 31706607

[18] Yang XJ Zhang J Chu P. “Pneumomediastinum secondary to foreign body aspiration: clinical features and treatment explorement in 39 pediatric patients”. Chin Med J (Engl). (2016) 129:2691–6. 10.4103/0366-6999.193450 27824001 PMC5126160

[19] Bai W Zhou X Gao X. “Value of chest CT in the diagnosis and management of tracheobronchial foreign bodies”. Pediatrics Int. (2011) 53:515–8. 10.1111/j.1442-200X.2010.03299.x 21129123

[20] Acharya K. “Rigid bronchoscopy in airway foreign bodies: value of the clinical and radiological signs”. Int Arch Otorhinolaryngol. (2016) 20:196–201. 10.1055/s-0036-1584293 27413398 PMC4942294

[21] Sriratanaviriyakul N Lam F Morrissey BM. “Safety and clinical utility of flexible bronchoscopic cryoextraction in patients with non-neoplasm tracheobronchial obstruction: a retrospective chart review”. J Bronchology Interv Pulmonol. (2015) 22:288–93. 10.1097/LBR.0000000000000203 26439016

[22] Seaman JC Knepler JL Bauer K. “The mean green popsicle: using cryotherapy to remove aspirated foreign bodies”. J Bronchology Interv Pulmonol. (2010) 17:348–50. 10.1097/LBR.0b013e3181f29e21 23168960

[23] Kazachkov M Vicencio A. “Foreign body removal is getting “cooler””. Pediatr Pulmonol. (2016) 51:886–8. 10.1002/ppul.23521 27378166

[24] Dalal PG Murray D Messner AH. “Pediatric laryngeal dimensions: an age-based analysis”. Anesthesia Analgesia. (2009) 108:1475–79. 10.1213/ane.0b013e31819d1d99 19372324

[25] Holzki J Brown KA Carroll RG. “The anatomy of the pediatric airway: Has our knowledge changed in 120 years? A review of historic and recent investigations of the anatomy of the pediatric larynx”. Paediatr Anaesth. (2018) 28:13–22. 10.1111/pan.13281 29148119

[26] Davis RJ Stewart CM. “Complete glottic obstruction by an unusual foreign body”. Otolaryngol Head Neck Surg. (2019) 160:935–6. 10.1177/0194599818824298 30645187

[27] Tamiru T Gray PE Pollock JD. “An alternative method of management of pediatric airway foreign bodies in the absence of rigid bronchoscopy”. Int J Pediatr Otorhinolaryngol. (2013) 77:480–2. 10.1016/j.ijporl.2012.12.010 23294930

[28] Singh JK Vasudevan V Bharadwaj N. “Role of tracheostomy in the management of foreign body airway obstruction in children”. Singapore Med J. (2009) 50:871–4. 19787173

[29] Batra H Yarmus L. “Indications and complications of rigid bronchoscopy”. Expert Rev Respir Med. (2018) 12:509–20. 10.1080/17476348.2018.1473037 29727208

[30] Youssef SJ Orbelo DM Sakata KK. “Dysphonia due to vocal cord injury after rigid bronchoscopy: a case study with 1-year bronchoscopic follow-up”. J Bronchology Interv Pulmonol. (2019) 26:e52–55. 10.1097/LBR.0000000000000587 31569103

[31] Lawlor CM Dombrowski ND Nuss RC. “Laryngeal Web in the Pediatric Population: Evaluation and Management”. Otolaryngol Head Neck Surg. (2020) 162:234–40. 10.1177/0194599819893985 31842676

[32] Zhang X Li WX Cai YR. “A time series observation of Chinese children undergoing rigid bronchoscopy for an inhaled foreign body: 3,149 cases in 1991-2010”. Chin Med J (Engl). (2015) 128:504–9. 10.4103/0366-6999.151104 25673454 PMC4836255

[33] Halwai O Bihani A Sharma A. “A study of clinical presentations and complications of foreign body in the bronchus - own experience”. Otolaryngol Pol. (2015) 69:22–28. 10.5604/00306657.1131145 25753164

